# Clozapine-Induced Myocarditis: Is Mandatory Monitoring Warranted for Its Early Recognition?

**DOI:** 10.1155/2014/513108

**Published:** 2014-01-23

**Authors:** T. A. Munshi, D. Volochniouk, T. Hassan, N. Mazhar

**Affiliations:** Queen's University, 385 Princess Street, Kingston, ON, Canada K7L 1B9

## Abstract

Clozapine is an atypical antipsychotic used for treatment resistant schizophrenia. Its potential to induce agranulocytosis is well known but it can also cause myocarditis. Clozapine is the only antipsychotic known to induce this side effect, typically early in the treatment, and literature is scarce on this condition. We are presenting a case report of a 21-year-old schizophrenic male who developed myocarditis within 3 weeks of starting on clozapine for his treatment resistant psychosis. We then aim to review some of the available literature and raise awareness among physicians as this condition can potentially be fatal if not detected early.

## 1. Introduction

We are presenting the case of a 21-year-old male with 2 years of ongoing psychotic symptoms not responsive to several trials of medication. He was started on clozapine and developed myocarditis on the 23rd day after initiation of treatment.

Clozapine is a tricyclic dibenzodiazepine derivative classified as an atypical antipsychotic. It is the treatment of choice for treatment resistant schizophrenia, defined as a lack of response to at least two antipsychotics after a trial of 6–8 weeks. Its advantages include low risk of extra pyramidal symptoms (EPS) and it has been shown to significantly reduce suicidal behaviour in patients with schizophrenia.

In terms of side effects, agranulocytosis and neutropenia are frequently recognized as serious ones and there are elaborate protocols to monitor and manage these. Cardiac side effects, including myocarditis, are perceived to be a more rare complication and there are currently no monitoring protocols. According to reports, more than 85% of the cases occur in the first 2 months and up to 75% within 3 weeks [1].

Myocarditis is an inflammation of the myocardium causing myocyte injury and can result in heart failure. Myocarditis is most often of viral etiology but it is also induced by several drugs and can be due to an autoimmune disorder [2]. Little is known about the pathophysiology of clozapine-induced myocarditis, but the mechanism is postulated to be a type 1 hypersensitivity reaction [3]. This condition has a variety of presenting symptoms. Many cases have a nonspecific “flu-like” presentation, including fever, shortness of breath, dry cough, and an elevated WBC [4]. There is frequently overlap with some symptoms of the “classic” hypersensitivity reaction, including fever, peripheral eosinophilia, sinus tachycardia, and a rash [2, 5]. However, there is no “classical” presentation of clozapine-induced myocarditis. Several authors have compiled tables of the most frequently reported symptoms [6, 7]. The most commonly reported symptoms are fever, tachycardia, and chest pain, and they occur in frequencies ranging from 32% to 49%. The most common abnormal investigations include a nonspecific “abnormal EKG” in up to 66% of cases, elevated troponins, and an elevated WBC. There are several suggested monitoring protocols [8].

The mainstay of treatment remains supportive [2, 9]. Mortality rates as high as 50% have been associated with clozapine-induced myocarditis [1, 3, 10, 11], a delayed diagnosis resulting in poorer outcomes.

The first case report of clozapine-induced myocarditis was published in 1980 [12]. Since then, a few more publications have looked into this problem, mostly from Australia [13]. Literature and case reports from other countries remain scarce.

The rates of occurrence appear to range from 0.015% to 0.188% [1, 3, 11, 14, 15]. One Australian review of 116 cases reported a rate of 1.2% among the patients on clozapine [4]. Some authors believe that the reported risk is likely to be an underestimate, given the wide range of presenting symptoms for this condition, making diagnostic identification challenging.

Indeed, the high variability of presenting symptoms, along with the high mortality rate, and the absence of known predisposing factors make it imperative, in our view, to raise more awareness among clinicians. We also suggest an approach to diagnosis and monitoring, based on our review of literature.

## 2. The Case

Our patient is a 21-year-old male diagnosed with schizophrenia following the onset of psychotic symptoms at age 19. The patient was experiencing auditory hallucinations and conversing with them and had persecutory delusions, somatic delusions, and delusions of passivity, as well as thought broadcasting, insertion, and withdrawal, and disorganized thought process. Organic etiology was excluded through examination and investigations. At various other times since the onset of his illness, he also experienced delusions of having special powers and visual hallucinations. There were no prominent mood symptoms except when he had insight into his illness and the awareness of the disruptive effect of his symptoms on his social functioning was causing him anxiety.

On family history, his brother had psychotic depression and his father had bipolar affective disorder type 1. This patient was abusing drugs such as alcohol, cannabis, and opiates such as codeine and oxycontin, prior to the onset of his symptoms. He eventually discontinued drug use but continued to use occasional alcohol and smoke cigarettes 1/2 to 1 pack a day. He resided in group housing and was followed by a community outreach treatment team.

For symptoms control, he was initially tried on Risperidone, then on Quetiapine XR, on Olanzapine, and finally on Aripiprazole. About a year into his illness, he experienced racing thoughts and irritability, and Valproic acid was added. He continued to experience the same psychotic symptoms and his mental status continued to show a formal thought disorder, affective flattening, delusions, and auditory hallucinations. Clozapine was initiated, starting at 12.5 mg, with view to titrate to 300 mg over the course of several weeks while monitoring side effects. The community outreach team monitored his vital signs on a daily basis and he attended weekly appointments with the psychiatrist to review and slowly escalate the dose according to protocol. The patient initially experienced only constipation and transient mild tachycardia and presented to the emergency department on day 6 of the treatment for a complaint of weakness, with normal vital signs and no other symptoms.

At baseline, the patient was a physically healthy male with no metabolic syndrome and no prior cardiac history. He has a documented allergy to sulpha drugs. His baseline investigations were within normal range, including EKG. His vital signs were within normal range. Before starting clozapine, his medications included Aripiprazole 25 mg qd, Valproic acid 500 mg bid, and Escitalopram 20 mg qd.

On day 23 following initiation of clozapine, the patient presented to the ER with complaints of a throbbing, pleuritic chest pain radiating to the throat, relieved by lying on the side or sitting up. He denied any shortness of breath. He had no nausea, no diaphoresis, and no dizziness. His clozapine was up to 50 mg in the morning and 75 mg at night; last dose adjustment was 2 days prior.

His vital signs showed tachycardia at 142 but no fever and the blood pressure was within normal range. He was alert and oriented. His EKG showed diffuse ST elevations with concave up slopes, mild PR interval prolongation, and a normal QT interval. His troponins were elevated at 0.383 (normal range 0–0.06). His WBC showed elevated neutrophils but no eosinophilia. D dimers were negative. Creatine kinase was very slightly elevated at 207. Chest X-ray was normal. Urine drug screen was negative.

He was diagnosed with myocarditis, clozapine was discontinued immediately, and he remained in the hospital overnight, followed by the cardiology team. He was then discharged on Ibuprofen, with cardiology followup and a scheduled outpatient transesophageal echocardiogram in 12 days. The results showed normal LVEF, normal systolic and diastolic function, normal valve morphology, and no pericardial effusion.

The patient was titrated up to an antipsychotic dose range of Quetiapine XR and maintained on Aripiprazole and Valproic Acid and remained compliant. He experienced relief of his psychotic symptoms after several weeks, although they never resolved completely.

## 3. Discussion

We have presented the case of a 21-year-old male with treatment resistant schizophrenia, no prior cardiac history and normal baseline investigations, who developed myocarditis on the 23rd day post-clozapine initiation. He was on a total dose of 125 mg of clozapine at that time, as well as on Valproic Acid, Aripiprazole, and Escitalopram. On presentation he has some of the previously described symptoms of myocarditis including tachycardia and chest pain but not others such as fever or eosinophilia. His symptoms resolved overnight with discontinuation of clozapine and supportive care and cardiac followup with echocardiography found no sequelae. Echocardiography is commonly recommended as the initial diagnostic evaluation tool of all patients with myocarditis [2]. Patients with chest pain only, without heart failure symptoms, almost always have a normal echocardiogram, as was the case for our patient. There is no consensus on routine screening for LV dysfunction prior to clozapine initiation. In addition, the absence of prior cardiac dysfunction has not yet proved to have any predictive value, as was the case with our patient.

In fact, there are no known risk factors for this condition. One case report suggested that physical exertion could be a risk factor [16]. Generally authors concur that there does not appear to be a dose dependent risk increase, as virtually all cases occur while the dosage is in the recommended therapeutic range between 100 and 450 mg a day. The absence of a dose dependent effect would also go along with the predominating causation hypothesis, which implies that drug-induced myocarditis is mediated by a hypersensitivity type reaction.

A number of other medications [2, 5] have been implicated in myocarditis, including lithium, thyroxine, and antibiotics such as sulfonamides and street drugs such as cocaine, and even ranitidine has been cited as a causation agent in one case report [17]. Concomitant valproic acid has also been found in several case reports of clozapine-induced myocarditis overall [4].

It would be interesting to investigate further to which extent concomitant administration of other drugs increases the risk of developing myocarditis if started on clozapine. It is interesting to note that our patient also has a documented allergy to sulpha drugs, especially keeping in mind the hypersensitivity reaction hypothesis.

Genetic susceptibility is deemed an unlikely contributor, but some researchers are still looking into it, especially given the predominance of case reports from Australia [13]. So far, looking into clozapine catabolism in Australian subpopulations has not confirmed genetic differences hypothesis [3, 18]. There have been recent genetic studies on genetic susceptibility to myocarditis, but of the autoimmune variety only [19, 20].

In addition to the absence of known risk factors, early detection is difficult because of the absence of specific symptomatology. Some symptoms are simply reminiscent of a flu-like illness, and benign transient tachycardia frequently occurs during treatment initiation. Given these difficulties and the high lethality potential, we believe monitoring this side effect should be firmly embedded in the protocols.

Our literature review identified several suggestions for improvements to current monitoring protocols [7, 14, 21–23]. Essentially they focus on keeping a high index of suspicion and early identification of symptoms. We would agree with the suggestion to incorporate cardiac markers such as troponins in weekly blood work, as well as a marker for acute inflammation, namely, CRP. Troponins are conclusively believed to be superior to CK for assisting in the diagnosis of myocarditis [2, 24, 25], with a high specificity (89%) but low sensitivity (35%). CK has no recommended role in screening [2]. CRP is a nonspecific early marker of acute inflammation; according to several studies [8, 22], CRP elevation occurs as one of the earliest signs, at the time of the most nonspecific symptoms, and predates troponin elevation by up to 5 days [22].

Combining troponins and CRP measurements, one study [22] has found a sensitivity of 100% for myocarditis identification. We would also recommend an EKG weekly for the first 4 weeks.

Some authors [6, 26–28] suggest that Type B Natriuretic Peptide holds promise for early myocarditis diagnosis. BNP is released from ventricles in response to increasing wall stress and is currently considered a good predictor of CHF. One case series of 5 patients with myocarditis secondary to clozapine [26] found that BNP was elevated at the time of diagnosis and decreased once symptoms resolved. This tool appears promising for future investigation in this context.

## 4. Conclusion

Clozapine is uncommonly associated with myocarditis, which can be fatal. It typically occurs within the 1st month of treatment initiation, in young, previously healthy patients with cardiovascular history and normal baseline investigations. There are no known risk factors. Initial suspicion is frequently low: this condition is uncommon and it can present with a wide range of symptoms, some of which are remarkably nonspecific, and others, such as tachycardia, as expected early in the treatment and are benign. These factors may in fact lead to underestimating the prevalence of this condition. We believe that additional awareness, increased clinical vigilance and patient education, and a more intense monitoring of this condition are warranted. Future directions for research could include validating additional monitoring parameters such as weekly troponins, CRP possibly BNP, to enable earlier detection of this condition.
